# Postgraduate learner perspectives on transforming learner handover to promote self-regulated learning

**DOI:** 10.1186/s12909-025-08557-x

**Published:** 2026-01-12

**Authors:** Allen Tran, Aaron Leblanc, Ian Epstein, Nabha Shetty, Caitlin Lees, Jenna MacGregor, Babar Haroon, Jorin LindenSmith, Robyn Doucet

**Affiliations:** 1 Department of Medicine, Dalhousie University, Halifax, Canada; 2 Department of Critical Care, Dalhousie University, Halifax, Canada; 3 Postgraduate Medical Education, Faculty of Medicine, Dalhousie University, Halifax, Canada; 4 Department of Anesthesia, Perioperative and Pain Medicine, Dalhousie University, Halifax, Canada

**Keywords:** Learner handover, Forward feeding, Postgraduate education, Learning plans

## Abstract

**Background:**

Many medical training models include discrete rotational blocks, which can be a barrier to the longitudinal assessment and coaching of learners. Learner handover is the sharing of information on a given learner’s progress between faculty supervisors and could aid in longitudinal feedback. Perspectives on learner handover by faculty supervisors and educational leaders vary. Learners’ perspectives on learner handover between faculty supervisors are lacking. This study aimed to obtain the perspectives of internal medicine postgraduate learners on learner handover practices and desired characteristics of formalized learner handover.

**Methods:**

Postgraduate learners in a single internal medicine program at a single center in Canada were invited to participate. This qualitative study used dyadic/triadic interviews conducted in a virtual format to collect data. A clinician-educator outside of the training program facilitated the interviews. Two team members conducted iterative, inductive analysis to develop codes and themes using thematic analysis. The final themes were generated after a series of reflexive discussions with our purposefully assembled research team of faculty and learners with varied experience and perspectives within medical education.

**Results:**

Seven of the 86 (8%) postgraduate learners chose to participate. Despite participating in learner handover, the learners did not identify the process as a known phenomenon. The identified benefits include longitudinal coaching, self-regulated learner development, and advanced knowledge of the strengths and weaknesses of the clinical teaching unit learners on rotation. The risks of biasing supervisors and learner anxiety were noted. To improve learner handover practices, learners suggest that learner handover should be learner-centric, have a minimal impact on the current assessment burden, standardized, and transparent. A model to implement learner handover that integrates these findings with the existing literature and promotes self-regulated learning is described.

**Conclusion:**

Postgraduate learners have similar perspectives on learner handover as other groups in medical education. A model for learner handover that leverages existing learner handover activity into a process for developing a self-regulated learner is created from these findings and the literature. This can inform postgraduate training programs on how to formalize learner handover practices to benefit the learner’s development and provide targets for faculty development.

**Supplementary Information:**

The online version contains supplementary material available at 10.1186/s12909-025-08557-x.

## Background

Competency-based medical education (CBME) is the current paradigm in postgraduate medical education in Canada [1–3] and other countries [4]. Programmatic assessment within CBME involves frequent, low-stakes assessments that emphasize assessments “for” learning rather than “of” learning: [5] measuring outcomes over time can highlight the strengths and weaknesses of a trainee, providing learners the opportunity to seek out specific learning opportunities [6]. In practice, achieving meaningful longitudinal evaluation has been challenging. Many postgraduate residency programs are organized into rotational blocks, meaning that learners work with numerous supervising faculty, each of whom has relatively little exposure to the learner. They are unable to build upon assessments and feedback that the learner has already received.

Learner handover, or forward feeding, is the sharing of information on a given learner’s progress between supervisors and is one potential solution to this issue of a lack of longitudinal and progressive evaluation [7]. Learner handover is a controversial topic in medical education, with arguments both for and against this approach [7–16]. Potential benefits include the early development of specific learning plans, feedback targeted at identified areas for improvement, the organization of advanced learning opportunities on the basis of strengths, teacher efficiency, and patient safety. However, this must be balanced against the potential risks to learner privacy and biasing supervisors [10, 13, 14, 17]. Many medical schools do not have official policies on learner handover [9, 12], so learner handover occurs informally and inconsistently [7, 9, 12, 15]. One study of 23 faculty members from 2 Canadian universities reported that all participants engaged in learner handover and advocated that it was a valuable educational practice [5]. 

Many of the educational-focused benefits of learner handover could further develop self-regulated learning skills by providing the learner with additional external feedback. Self-regulation refers to self-generated thoughts, feelings, and behaviors that are oriented toward attaining goals [18]. The use of feedback and learning plan development has influenced self-regulated learning among medical learners [19]. Informal and inconsistent learner handover that excludes the learner is a missed opportunity for the development of self-regulated learning skills.

Prior research on learner handover has highlighted the perspectives of faculty members [5, 7, 10, 13–15]. There has been large interest in learner handover between undergraduate medical education to postgraduate medical education [6, 9, 12, 15, 20, 21]. Comparatively, the learner perspective on learner handover occurring between faculty supervisors is less understood [22, 23]. Few studies have focused on medical students’ [22, 23] or postgraduate learners’ [23] views on learner handover practices between faculty members and the acceptability of this practice despite this group being the intended beneficiary of learner handover that occurs in an educationally positive manner. Postgraduate learners work for years alongside a smaller group of supervising faculty and potential future colleagues, so concerns about negative bias with learner handover may be amplified for postgraduate learners. However, this same circumstance may provide even greater potential benefits from learner handover. The supervising faculty may feel a greater sense of responsibility and investment in their postgraduate learners and be more willing to engage in learner handover practices that further the learner’s development.

Learner handover is already widely occurring in an informal and inconsistent way [7, 9, 12, 15], and leveraging this practice to improve learner feedback from faculty supervisors presents an opportunity in medical education. The learner should be at the center of educational activities, but there is minimal information about the perspectives and experiences of postgraduate learners on learner handover that occurs between faculty supervisors. Our study aimed to elucidate the perspectives of internal medicine postgraduate learners on learner handover and the desired characteristics of a formalized learner handover process.

## Methods

### Setting, study design, and epistemological framework

This qualitative study was conducted at Dalhousie University in Halifax, Canada. The target population was internal medicine postgraduate learners (postgraduate year [PGY] 1–3). This study population was chosen as many of the authors completed internal medicine training and are thus well situated to understand the complexities and nuances of the training environment. This study was approved by the Dalhousie University research ethics board.

Our research questions are best answered through qualitative methodology, as they seek an understanding of viewpoints [16]. We used social constructivism as our epistemological framework because we are interested in the lived experiences and values of postgraduate learners in the context of complex social interactions involved in learner handover processes and how that results in a unique constructed reality for each person [24, 25]. The social interactions between a learner and another learner, faculty supervisor(s), program director, competence committee, academic advisors, and/or division/department head are examples that illustrate the potential combinations of relationships that could influence one’s view of learner handover. Our study team members hold many of these roles, and the social constructivist framework allows the researcher to be a more active participant in the research and interpretation of the data [26]. 

### Participant recruitment

All internal medicine postgraduate learners were invited to participate in dyadic/triadic interviews, with calls for participation through a presentation at their academic half-day, followed by mass emails in March 2022 and October 2022. There were no exclusion criteria. Participation was voluntary, and written informed consent was obtained. Coercion was mitigated by having a research assistant handle recruitment, organize dyadic/triadic interviews, and deidentify the data.

### Data collection

The data were collected through dyadic/triadic interviews and informed by a semi-structured topic guide (appendix 1). This method of data collection was selected for multiple reasons [27, 28]. First, multiple viewpoints and diverse experiences can be obtained in an efficient manner compared with individual interviews. Second, learner handover may not be an experience that every participant has personally undergone; a multi-person interview allows these participants to hear directly from peers who have the lived experience of learner handover rather than base discussion on theoretical explanations. Third, a rich discussion can occur in a group that includes contrary views and personal reflections compared with single interviews or surveys. Fourth, it can be logistically difficult to gather many postgraduate learners together at once for a focus group interview consisting of 6–10 individuals due to varied demands from clinical rotations, on-call requirements, and their personal lives. Dyadic/triadic interviews can be a more pragmatic approach to obtain qualitative data. Given the potential drawback of dyadic/triadic interviews generating discomfort for some participants when discussing personal experiences, an individual interview was also offered to all potential participants to maximize data collection.

The topic guide followed priority topics identified by our research team based on experience with learner handover and prior literature. The topic guide was piloted with faculty members outside of our research team to obtain feedback on the clarity of the questions and the need for potential revisions. Gender and postgraduate year (PGY) data were collected at the time of the dyadic/triadic interviews.

A clinician-educator outside of the training program was chosen as the dyadic/triadic interview facilitator (R.D.) to increase the likelihood of authentic data, as this research team member was not involved in supervision or assessment of the participants and did not have any prior relationships with the participants. The dyadic/triadic interviews were 60 min in duration and were conducted virtually with Microsoft Teams. The interviews were recorded, anonymized, and transcribed verbatim. The anonymized transcripts were shared with the investigators for data analysis. A $20 gift card was provided to the participants.

### Data analysis

We intended to use grounded theory methodology because of the need to understand the social processes that answer our research questions and to generate theory grounded in the practical experience of the study participants [29]. This theory aligns with our social constructivist theoretical framework [24]. However, we experienced a lower than expected recruitment rate which prevented us from following all principles of grounded theory methodology, including theoretical sampling based on the initial data. Due to this limitation, we adopted a thematic analysis approach [30]. We analyzed dyadic/triadic interviews transcripts via a constant comparison and iterative approach. Two individual members of our team (A.L., A.T.) initially independently coded the data through inductive analysis. These two coders then had frequent discussions to resolve disagreements, iteratively review the data and re-code, and derive emerging themes from the codes. The entire research team subsequently reviewed the findings and met multiple times to discuss the identified themes while having a reflexive discussion to determine a final set of themes. We used NVivo 12 (Release 1.7.1) for coding.

### Reflexivity

We purposefully assembled our research team based on their various levels of experience and perspectives. Membership included current/past residency program directors (A.T., A.L., B.H., I.E., R.D.), past competence committee chairs (A.L.), a postgraduate dean (B.H.), academic center faculty supervisors of varying experience (A.L., A.T., B.H., I.E., R.D.), community site faculty supervisors (J.L.), graduates within the last 2 years (C.L.) and a postgraduate learner (J.M.). We involved diverse team members to provide potentially richer and more diverse perspectives on learner handover [31]. During the meetings, we reflected on our potential biases related to our roles and experience in medical education, such as our personal views on the intent, perception, and utility of learner handover. Having a diverse group allowed us to mitigate these biases by regularly challenging one another’s interpretations of the data in these discussions. Both researchers leading data analysis (A.L., A.T.) have advanced training in medical education, hold postgraduate educational leadership positions, and have completed internal medicine postgraduate residencies.

## Results

Seven of the 86 (8%) postgraduate learners chose to participate. Five participants (71%) were female-identifying. Two of the participants were at the PGY1 level, three were at the PGY2 level, and two were at the PGY3 level. Three interviews were conducted between June 2022 and March 2023, with 2 to 3 participants each. The dyadic/triadic interviews were composed of a mix of PGY level participants. There were no requests for individual interviews.

We identified seven themes organized into two categories. Within the first category, learner perspectives on learner handover, themes included: ‘informal process and culture’, ‘learner development’, and ‘learner stress’. Within the second category, the desired characteristics of a formalized learner handover process included the following themes: ‘standardization of the process’, ‘minimizing administrative burden’, and ‘learner-centric’.

### What are the perspectives of internal medicine postgraduate learners on learner handover?

#### Informal process and culture

The participants were able to identify learner handover once the concept was openly discussed, and they perceived or assumed it was ingrained in the culture of training:

“When I was a medical student or a junior resident, to be honest, I don’t know that I gave it a whole lot of thought. I just thought that’s part of the process for what goes on.” (Participant 6).

There was also recognition of their dual role as a learner and a supervisor in a hierarchical framework. Their role and participation in learner handover as supervisors were then brought to the forefront. Participant 2 recalled: “Very informal about myself, but I have been on the other side of that. Handing information on medical students or junior residents over to other team members.”

The participants’ perspective was that the learner handover focused on current clinical performance. They did not perceive that there was a clear structure or objective of learner handover between faculty supervisors. Notably, the learner’s specific goals were not identified as a component of the learner handover.

#### Learner development

The participants described multiple ways that learner handover could further their development. Learner handover provides an opportunity for longitudinal coaching across supervisors. Participant 1 stated, “Your supervisor might change from week to week. It might be useful to hand over sort of what could be improved just so that there’s some continuity in terms of your own development.” As well, there is the potential to facilitate the achievement of specific learner goals or interests over time. The development of the skills necessary for self-regulated learning, through reflection on past feedback, was discussed as a potential benefit with a dedicated learner handover process.

“And then if when you start your next rotation or the next week, if you meet with your staff at the beginning … and then talked about, you know, things you wanna work on or things you find are more difficult. Then, you can kind of draw on the feedback that you’ve gotten from previous evaluations and share that with your staff at the beginning of the week or the month. That kind of makes the learner involved and gets the handover indirectly to the staff.” (Participant 5).

Learner handover could improve efficiency through providing a “head start” for the next supervisor to help the learner with specific areas for improvement.

“If someone just kind of gave me a heads up. Like hey, this clerk needs a lot of help with their history and physicals, and you should probably take more of an effort to have them observe you. That would help me help them.” (Participant 4).

#### Learner stress

Discomfort with the thought of being discussed can occur even when the learner knows it is positive handover.

“…one of the staff on one rotation apparently was friends with someone that I was having a meeting with. And then they said, ‘Oh, I heard really good things about you on your whatever rotation,’ and I was like oh that’s really weird. Like you guys are friends and you were talking informally about me.” (Participant 5).

Concerns arose surrounding the risk of bias and the effect the provider of learner handover could have on the receiver. An incomplete picture of the learner can also come through in learner handover, based on the context of that interaction with the supervisor.

“I suppose I worry about perceptions. Getting passed forward, both like positive perceptions that carried out momentum, but also negative perceptions of one person, and I feel like medicine is significantly trustful and hierarchical. … While I think it could be a good thing, I think it depends on the content of that feedback and who’s saying it.” (Participant 2).

### What are the desired characteristics of a formalized learner handover process?

#### Standardization of the process

There is a desire for learner handover processes to be made explicit, standardized, and documented. This could increase learner engagement. Participant 5 explained, “A session explaining what the intent is, that it’s for the benefit of the learner to help them get better, and it’s not to help with evaluation or to create biases.” Having a structure for learner handover through handing over only direct observations and avoiding judgment statements in the handover were thought to increase the utility while decreasing the risk of bias.

#### Minimizing administrative burden

A concern arose regarding learner handover practices potentially interfering with other assessments required for progression in a training program. Prior to implementation, determining who would be responsible for gathering the information needed for learner handover and how to prevent evaluation fatigue were important considerations. This was highlighted by participant 2’s concern: “I would be worried that this would fall upon the learner to chase.”

#### Learner-centric

While there are multiple possible beneficiaries of learner handover, it was thought that learner handover processes should primarily benefit the learner and that the learner should be centrally involved in goal setting and discussions. The development of self-regulated learning skills and reducing anxiety were noted as benefits.

“It would be interesting to see how the learner says, ‘I am a resident who is good at A, B and C, but what I need feedback on is like X, Y, Z and that’s what I hope to achieve in the next year.’ … you’re saying this is where I need help.” (Participant 2).

## Discussion

We conducted a qualitative study utilizing dyadic/triadic interviews with postgraduate medical learners to understand their perspectives on learner handover and how it could be best implemented as a formalized medical education practice. Our study participants often contributed to informal learner handover, although it was unstructured, inconsistent, and often did not center on the goals of medical learners. The participants viewed learner handover as a tool for learner development through longitudinal coaching, facilitating learner-specific goals, and development of self-regulated learning skills. However, learner stress also comes along with learner handover practices through the concerns of biasing future supervisors and the discomfort of not knowing when they are being discussed.

This study contributes the perspective of postgraduate learners to prior research, which has emphasized the perspectives of faculty supervisors and medical students. Our findings regarding the potential benefits of longitudinal coaching, the development of self-regulated learning skills, and benefits to the supervisor [5, 10, 12, 13, 22, 23] and the potential risks of biasing a future supervisor and learner anxiety [5, 7, 12–14, 22, 23] are consistent with prior research. The views were similar whether the target population was learners [12, 22, 23], faculty [5, 12, 13], or educational leaders [7, 10, 12, 14]. The postgraduate learners we interviewed did not understand learner handover as a defined practice prior to the study, suggesting that their views were uninfluenced by knowledge of the literature.

Uniquely, when discussing how to best operationalize learner handover, the suggestions from the postgraduate learners in this study resemble principles of high-quality feedback [32, 33]. This study took place during the third and fourth years after the transition of this residency program to a CBME framework. There have been numerous reports of the unintended consequences of this transition leading to reduced feedback quality and frequency [34–36]. A large Canadian postgraduate learner survey confirmed the issue of the administrative burden of obtaining and monitoring CBME assessments being placed on learners [35]. A learner handover process adding to the assessment burden on learners was a uniquely prominent concern in our study, and the effects of CBME may have influenced these views.

**Fig. 1:**
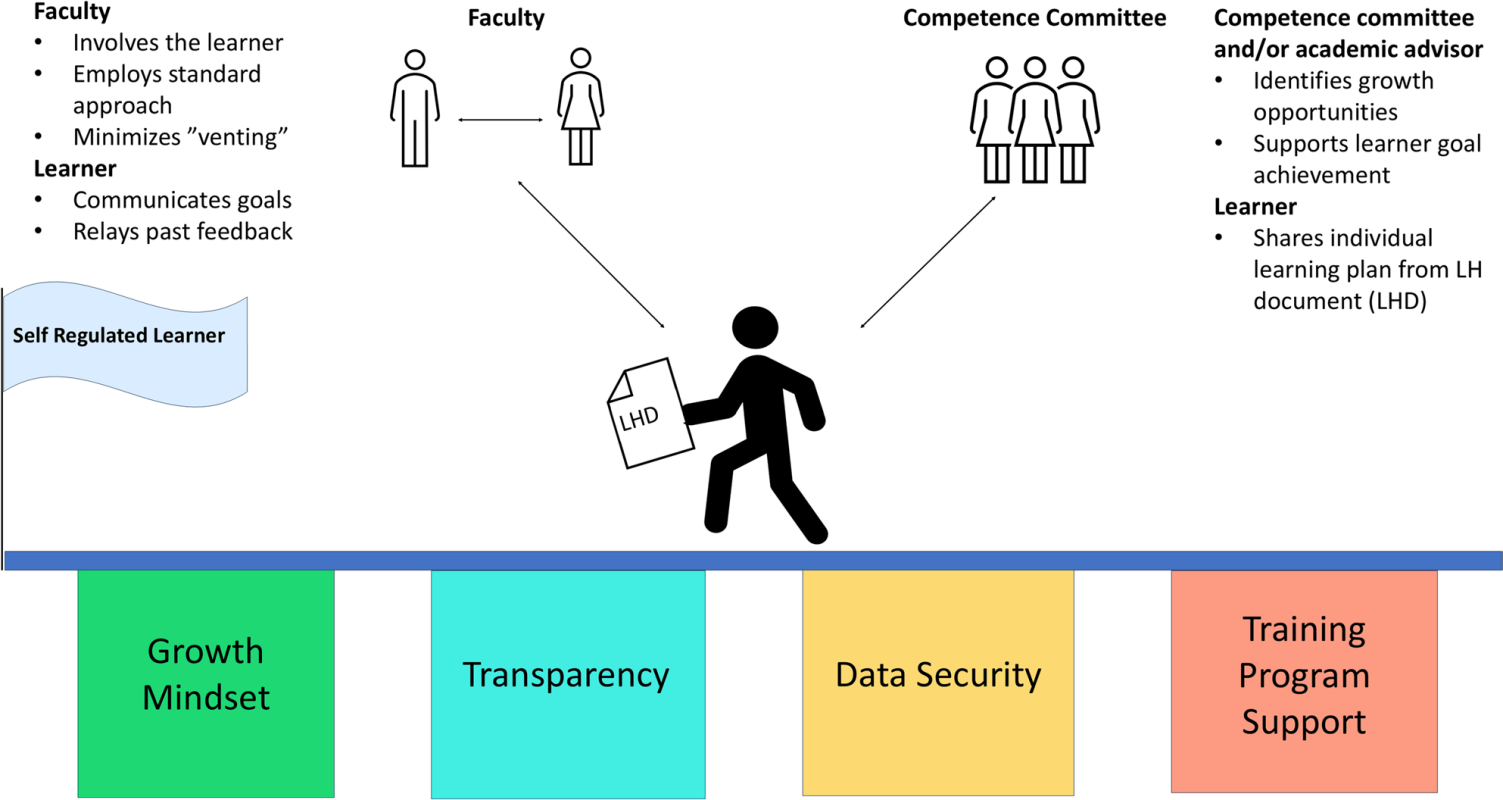
A model for learner handover LHD denotes learner handover document

Based on our findings, prior literature, and our research team’s personal and professional experience, we propose a learner handover model that is learner-centric and promotes self-regulated learning (Fig. 1). Guiding principles, supported by this study and the literature on learner handover, are that a best-practice learner handover model requires transparency, standardization [9, 12, 13, 22, 23, 37], and should foster continued improvement of the learner (Table 1) [5, 7, 9, 10, 12, 21, 22, 37]. Another similarity between learner and faculty perspectives on ideal learner handover implementation is the need for learner involvement in determining the content that will be handed over from faculty-to-faculty [6, 9, 37, 38]. 

**Table 1:** Principles of the model for learner handover

Guiding Principle	Rationale	Representative quote from our study
Transparency	Contributes to a growth mindset culture and eases learner distress regarding how the learner handover will be used. Builds trust between the learners and the educational program.	“A session explaining what the intent is, that it’s for the benefit of the learner to help them get better, and it’s not to help with evaluation or to create biases.” (Participant 5)
Standardization	Lowers the risk of bias and learner handover that does not have an educational purpose.	“I think if it’s a standardized thing like you mentioned… Then I can see that being a helpful thing and this is what they’re strong and this is what they’ve been working on, etc.” (Participant 3)
Continued improvement of the learner	The focus of learner handover should be on the learner’s development in a training program. This will, in turn, improve clinical care.	“…think having somebody who’s already seen you, who knows, you know, some of your strengths and weaknesses and able is able to hand that over to somebody who’s gonna oversee you, teach you for the next couple of weeks or week or whatever it is and for them to be able to hit the ground running knowing that you want to work on this or there’s opportunities to do like one-on-one teaching on physical exam because you don’t feel strongly about that.” (Participant 6)
Learner involvement	Co-creation of the learner handover between the learner and faculty promotes self-reflection and lowers learner stress through demystifying the practice.	“I do wonder if, like we found out or there was some way for us to know about what was exactly handed over. That might be more reassuring.” (Participant 7)

A psychologically safe environment promotes a growth mindset, which in turn is needed for learners to engage with learner handover. Achieving this engagement requires a shared mental model between learners, faculty supervisors, competence committees, and educational leaders. Shaw et al. reported how learners experience performance fatigue and anxiety, which is amplified with the prospect of learner handover occurring in a negative fashion [23]. Faculty development sessions can support redirection of the informal learner handover that is already occurring into a positive, tangible benefit for the learner. As noted by our participants, building trust through information sessions with the learners to explicitly describe the intent to benefit the learner’s development through the learner handover process would increase learner engagement. As noted in our study, our participants were not aware of the term of learner handover but recognized the practice. It is crucial to explicitly define the term and describe the process with all learners and faculty members to ensure there is a shared mental model.

Self-regulated learning skills are integral to responsible physician practice, and such skills can be encouraged in learners by supporting the development of an individualized learning plan. A co-created learner handover document may be a tool to facilitate this type of learner self-reflection while simultaneously permitting faculty supervisors to feed forward information. This is supported by our findings as the participants noted how learner handover can facilitate the development of self-regulated learning and achievement of targeted learning needs. Academic advisors or competence committees can provide mentorship and other possible learning goals based on all assessment data. A similar document has been suggested by others [38]. An educational quality improvement study to improve the development of learning goals during an inpatient rotation included a similar process [39]. In practice, data for the learner handover document could be filled during feedback sessions between a learner and supervisor. Academic advisors or competence committees could also have standing sections in their reports to highlight possible learning goals, which is then provided to the learner to consider entering in the learner handover document. The learner can also engage with the learner handover document at any time when reflecting on their experiences (e.g. at the end of a clinical day or upon reviewing their assessment data) and contribute self-identified learning goals to the document.

An effective learner handover document needs to be structured, concise, electronically filled, and accessible. Data security and privacy of such a document is required to maximize psychological safety. We recommend formats that may integrate with existing learner management systems or applications that could be used on mobile devices. Timeliness and ease of completion are important considerations given the concerns raised in this study about administrative burden with new interventions.

Control of the learner handover document is a key component of our model. Owing to the concern of biasing future supervisors, we suggest that the learner has sole access to the learner handover document. The learner would then communicate the information they intended to share with their upcoming rotation supervisors. This may help reduce stress learners experience when they are unaware of the conversations taking place between supervisors, as noted in our study. The satisfaction noted by the postgraduate learners in the study by Reed et al. [39] involved handing over learning goals that were purely from the learners themselves. Allowing the learner full control can be a mediating transition step that reduces the concern of learner handover documentation leading to unfair assessment until the not yet realized culture of a growth mindset becomes prevalent [35]. We acknowledge that this approach has the risk of the learner not disclosing significant areas for improvement that may affect patient safety. We believe that the current practice of the competence committee reviewing multiple assessment data points would mitigate this potential risk. Meetings with the academic advisor, co-creation of the learner handover document, and developing self-regulated learning skills could also help mitigate this risk.

Thus, our model (Fig. 1) depicts a learner, at the center, moving towards being self-regulated. The learner is using the learner handover document, as described above, that is informed from dialogue between the learner and faculty, the competence committee, and an academic advisor. This learner handover process is supported by the pillars of training program support, data security, transparency, and a growth mindset.

This model does not directly address informal learner handover. Practically, we do not believe this is possible to eliminate. The hope is that the mindsets of the faculty supervisors will be changed with the implementation of this model and faculty development regarding the issues of informal learner handover. It is prudent to highlight the issues surrounding learner handover that is used as a “venting” session [5, 23] and how prior performance information can influence future ratings [8, 11, 40, 41], even if only stating that a learner is above or below average [42]. 

Our study had limitations. This was a single center study of volunteer participants from an internal medicine postgraduate residency program, which may have limited the diversity of experiences and perspectives. While there was participation from all 3 years of the residency program, there was a small number of participants. The low participation rate may have been related to the study period occurring during the COVID pandemic when internal medicine wards had high patient volumes and significant postgraduate learner burnout rates. In addition to the study occurring during the COVID pandemic, it also occurred during the early phase of the transition to a competency-based education framework which was met with significant learner burnout [43]. The stressors of this transition, the pandemic, and the high patient volumes may have affected the responses of our participants. Our findings may have been different if the cohort came from a procedural residency program, such as general surgery. However, the perspectives of the participants correlated with existing literature on learner handover from many different contexts. The low participation rate also limited our ability to explore the learner insights in greater depth and breadth, with low confidence in achieving data and theoretical saturation. We are unable to identify whether views on learner handover differ based on training level or other factors owing to the small sample size and our prioritization of anonymity. While one-on-one interviews were offered, no participant opted for this method. This may have occurred due to the participants feeling they were able to contribute well in the dyadic/triadic format as compared with a larger focus group.

There are other potential next steps that could be undertaken by a future study to address our limitations and further understand the learners’ perspectives on learner handover. Using a questionnaire with open ended questions to explore learner perspectives on learner handover and a preferred method of implementing a learner handover process would increase the potential for broader input from learners. Feedback on the various components involved in our model would provide helpful insights. This questionnaire could also obtain insightful data such as frequency of the subject or the provider of learner handover. Another way to increase participation would be to broaden the study group to multiple sites and/or multiple different postgraduate specialty training programs. Data collection could be further increased through offering the ability to provide anonymous digital diaries over time regarding their reflections on learner handover as they occur.

## Conclusion

Our study provides further empirical evidence on the perspectives of postgraduate learners on learner handover practices that occur between faculty supervisors and provides a voice for this underrepresented group that should be the focus of learner handover when used as an educational tool. Numerous studies suggest that learner handover should be learner-centric, which places emphasis on ensuring that the benefit to the learner is prioritized. We have described a model that is synthesized from our findings and existing literature that refocuses learner handover into an exercise of developing a self-regulated learner. Postgraduate training programs can use this model to inform efforts to formalize learner handover practices into an intervention that supports learner development.

## Supplementary Information

Supplementary Material 1. Appendix 1 – Semi-structured topic guide used in the dyadic/triadic interviews.

## Data Availability

The data supporting this study’s findings are not publicly available to protect participant identity. However, upon reasonable request, deidentified data are available from the corresponding author.

## Abbreviations

CBME: Competency–based medical education
PGY: Postgraduate year
