# Supplementary material for: Postgraduate learner perspectives on transforming learner handover to promote self-regulated learning
Source: BMC Med Educ. 2026 Jan 12;26:223. doi: 10.1186/s12909-025-08557-x (PMC12888501; doi:10.1186/s12909-025-08557-x)
Supplement: Supplementary file 1 — Supplementary Material 1. Appendix 1 – Semi-structured topic guide used in the dyadic/triadic interviews. [file 12909_2025_8557_MOESM1_ESM.docx]

Appendix 1 - Focus group script

**Introduction:**

Thank everyone for joining and their participation.

Confirm that each participant has read the consent form, understands it, and has provided written consent to being recorded and the possibility of using quotations in our publication that will not be attributed to them. Inform them that they can withdraw their participation and data up to the end of the focus group. After that, the transcription will de-identify them so it will be impossible to remove what they've said.

Once consent has been confirmed, then gather the information about their postgraduate year.

Strongly encourage everyone to maintain confidentiality of the focus group discussion. Discuss the measures that we are taking to maintain confidentiality and privacy (only the research assistant [non-clinician] and yourself will have any identifying information of the participants, the data will be de-identified in the transcript, only the de-identified data will be available to the other researchers, the recording will be deleted as soon as the transcript is completed). Mention that we cannot control if the current participants break confidentiality and privacy of the participants though.

**Questions:**

Who has heard of learner handover or forward feeding? Describe what it means to you.

-If they have nothing, then learner handover is the sharing of information about learners between faculty supervisors, who may or may not be evaluating the learner

Has anyone experienced this before? Either as the learner or as the supervisor discussing a learner.

What are the group's thoughts on learner handover? (Good/bad, useful/harmful, any benefit)

-Who does it benefit and how? (faculty, learners, program director, patients, etc.)

Ask the group to consider the contradicting perspective if there has been predominately one view on learner handover

If at all, how could the process of learner handover be constructed so that it maximizes the educational and training experience of residents?

-Ask them to consider the players in their training (program director, academic advisor, general faculty, informal/formal mentors, more senior trainees, peer trainees, program directors of their potential future subspecialty).

-Are upfront policies needed?

-What aspects of learner handover do you think should be included or standardized? Why?

-What aspects of learner handover do you think should absolutely not be included? Why?

**Conclusion:**

Thank them again. Inform them that they still have a chance to have their data removed from the study. They can do this by staying on to let the facilitator know or emailing the research assistant who is the one who will be doing the transcription.

Also inform them that they could have a one-on-one interview if they wanted to discuss this topic further in that format. Contact the research assistant, Vanessa Higgins, ([vannessalhiggins@gmail.com](mailto:vannessalhiggins@gmail.com)) to set this up.

If we don’t have participation from all 3 years of training, ask the participants to please share the information about this study with their peers and to email the research assistant to participate.

Provide info to them regarding resources or support in case this discussion evoked emotional or psychological distress. This would include the Resident Affairs Office, the employment and family assistance program provided by MarDocs, and the professional support program provided by Doctors Nova Scotia.

The attendance list will be confirmed with the research assistant, who will then contact the participants to get the $20 gift card as a token of thanks.
